# Research on Stress–Strain Detection in Iron Specimens Using DIC and ACSM Techniques

**DOI:** 10.3390/s26154834

**Published:** 2026-07-31

**Authors:** Guangyong Yang, Zijing Chen, Zhengqiang Lei, Rui Li, Yanbing Wang

**Affiliations:** 1 PipeChina Institute of Science and Technology, Tianjin 300450, China; 2023216011@student.cup.edu.cn (Z.C.);; 2 China University of Petroleum (Beijing), Beijing 102249, China

**Keywords:** stress, strain, DIC, ACSM, inverse magnetostriction, NDT, COMSOL

## Abstract

Stress–strain detection serves as a common inspection technique in engineering integrity management, enabling the prediction of the health status and remaining service life of materials or structures. This study systematically investigates the performance and application effectiveness of Digital Image Correlation (DIC) and Alternating Current Stress Measurement (ACSM) technologies in the field of stress–strain detection. Based on the inverse magnetostriction effect and Maxwell’s equations, an ACSM detection system was developed, achieving the conversion of stress signals into electromagnetic signals. Simultaneously, DIC technology combined with a high-resolution binocular vision system was employed to realize non-contact measurement of full-field strain distribution. Finite element analysis using COMSOL V6.1 software was conducted to simulate the stress–strain distribution of ferrous specimens under axial tensile load, identifying the range of the gauge section with uniform stress–strain distribution. An experimental platform was established to perform tensile tests on flat specimens. The results demonstrated that the stress detection error of the ACSM system was less than 42.93 MPa, the strain measurement error of the DIC system was below 2.14722 × 10^−4^, and the stress inversion error was less than 31 MPa. A comprehensive comparison indicates that DIC technology offers superior performance in measurement accuracy and resolution, while ACSM technology provides advantages in operational convenience and rapid response, making it suitable for rapid screening in industrial settings.

## 1. Introduction

Stress–strain detection holds significant importance in engineering applications such as railways and pipelines. Under certain extreme conditions, the tensile stress induced by residual stresses can reach 1300 MPa, concentrated within the surface layer of materials [1]. Regular detection of the stress–strain state of materials provides quantitative basis for predicting material service life, evaluating structural integrity, and assessing reliability [2,3].

Stress measurement techniques have evolved into dozens of methods over the years [4], which can be broadly classified into two categories: destructive and non-destructive testing (NDT). Destructive methods include hole-drilling, layer-by-layer milling, and sectioning [5]. These methods can quantitatively calculate residual stresses with high accuracy, but they cause significant damage to the test object. Non-destructive methods include radiographic testing, ultrasonic testing, alternating current field measurement, and magnetic memory testing [6,7]. NDT techniques convert stress–strain signals into measurable signals such as magnetic and electrical signals through various physical effects, thereby achieving indirect stress–strain detection. Since NDT can achieve high-precision, repeatable measurements of internal stress–strain states without compromising the structural integrity of the test specimen, it has become an important research direction in stress–strain detection at present.

The Alternating Current Stress Measurement (ACSM) technique [8,9,10] evolved from the Alternating Current Field Measurement (ACFM) defect detection technique [11,12,13,14,15]. It achieves the conversion of stress signals into electromagnetic signals through the inverse magnetostriction effect and Maxwell’s equations. In 1998, Chen and Brennan formally proposed the concept of ACSM technology and established the first uniaxial stress theoretical model by integrating the half-space electromagnetic induction theory with the stress-magnetization constitutive relation [16], marking the extension of this technique from defect detection to quantitative stress measurement. Since then, the magneto-mechanical coupling theoretical framework of ACSM has been continuously refined: Zhou and Dover developed an analytical electromagnetic induction model for anisotropic half-space, elucidating the regulation mechanism of stress-induced magnetic anisotropy on detection signals [17]; Han et al. [18] constructed a biaxial stress characterization model under orthogonal magnetic fields, enabling multi-component decomposition under plane stress states. In recent years, researchers have further introduced the J-A magnetization theory and thermodynamic constitutive models [18,19,20], establishing magneto-mechanical coupling analytical methods adapted to combined loading conditions, thereby continuously improving the quantitative transfer relationship between stress and electromagnetic signals. Relying on the progressively matured theoretical foundation, extensive R&D work has been carried out on ACSM probes and hardware systems, including single excitation-pickup coil configurations and orthogonal differential coil designs, along with iterative upgrades in excitation signal generation, weak signal conditioning, and data acquisition systems. These developments have steadily enhanced detection sensitivity, anti-interference capability, and operational adaptability [21,22,23,24,25,26,27,28], and the technology has gradually been applied to stress detection scenarios in oil and gas pipelines, rail transit, and other fields.

Although ACSM technology has formed a relatively complete system at both theoretical and hardware levels, the quantitative accuracy and engineering application effectiveness of such electromagnetic stress inspection equipment are highly dependent on calibration tests and performance verification conducted under laboratory conditions. Current ACSM system calibration mostly uses single-point measurement data as the true-value reference, which cannot fully reflect the spatial distribution characteristics of the stress field, nor can it systematically quantify the spatial distribution pattern of detection errors. This limitation hinders further improvement of detection accuracy and the definition of engineering applicability boundaries. To address this issue, this study proposes a technical approach that uses DIC full-field references to assist ACSM stress detection: a self-developed ACSM stress detection system is constructed, combined with a high-resolution DIC detection platform; through finite element numerical simulation and axial tensile tests, the detection accuracy, error distribution patterns, and application boundaries of the two techniques under elastic tensile loading are quantitatively compared; and the DIC-derived full-field strain distribution results are used to provide data support for ACSM point selection and system calibration, offering a feasible technical path for integrity inspection of ferromagnetic engineering structures.

## 2. Principles and Methodology

### 2.1. ACSM Stress Detection Technology

#### 2.1.1. Technical Principle

**Figure 1:**
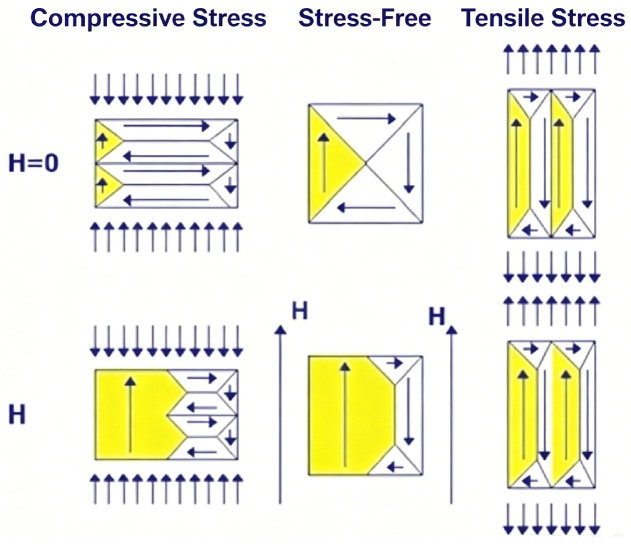
Schematic diagram of the inverse magnetostriction effect.

The inverse magnetostriction effect, which originates fundamentally from magneto-elastic coupling, describes the response of a ferromagnetic material’s magnetization state to externally applied mechanical stress. At the microscopic level, this coupling drives the reorientation of magnetic domains along the direction of the applied stress, accompanied by the migration and twisting of domain walls. Such stress-induced domain-wall evolution inevitably gives rise to changes in domain-wall thickness—an intrinsic consequence of the altered magnetic anisotropy. At the macroscopic scale, these microstructural rearrangements are reflected in observable variations in the material’s magnetic permeability, as illustrated in Figure 1. Specifically, tensile stress increases the permeability along the loading direction, whereas compressive stress decreases it [16,17,18,19,20].

According to Maxwell’s equations, electric currents and changing electric fields generate magnetic fields, while changing magnetic fields produce electric fields in their surrounding space. When an alternating electric field is applied to the excitation coil, it generates a primary magnetic field in space. This magnetic field acts on the ferromagnetic specimen whose stress is to be measured, inducing an eddy current field on the specimen surface. The changing eddy current field further produces a secondary magnetic field opposing the direction of the primary field, which impedes the original magnetic field variation and alters the impedance and induced voltage of the detection coil. Consequently, the impedance and induced voltage signals of the detection coil contain stress information.

#### 2.1.2. System Composition

**Figure 2:**
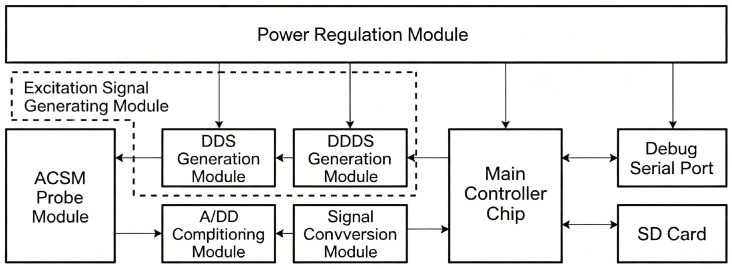
Hardware structure diagram of the ACSM system.

The ACSM stress testing system comprises several main components: a power supply voltage stabilization module, an excitation signal generation module, a probe module, a signal conditioning module, an A/D conversion module, a main control chip, a debugging serial port, and an SD card. The hardware structure diagram of the system is shown in Figure 2.

**Figure 3:**
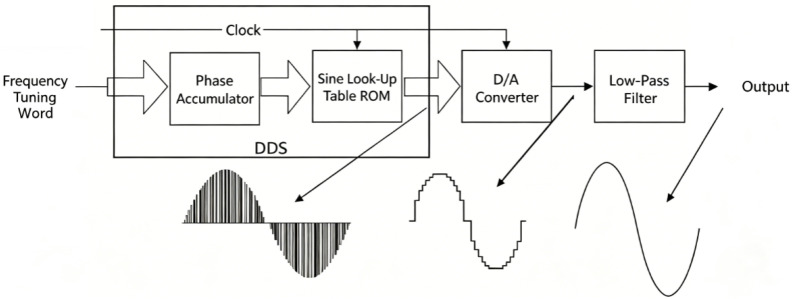
Internal functional structure of DDS.

The excitation signal generation module includes a Direct Digital Synthesis (DDS) generation module and a power amplification module. It provides a sinusoidal excitation signal of a certain frequency to the excitation coil of the ACSM probe module, inducing a uniformly varying magnetic field on the specimen surface. DDS technology is based on the time-domain sampling theorem, sampling sinusoidal signals to obtain a large amount of phase information, which is then analyzed and calculated to derive corresponding amplitude information. After smoothing and filtering, the desired signal output is achieved [11]. The internal functional modules of DDS mainly include a phase accumulator, a function lookup table, and a digital-to-analog converter, as shown in Figure 3.

Since the sinusoidal excitation signal provided by the DDS generation module has insufficient driving capability to directly drive the excitation coil, a power amplification module is required to amplify the excitation signal to enable load-driving capability.

**Figure 4:**
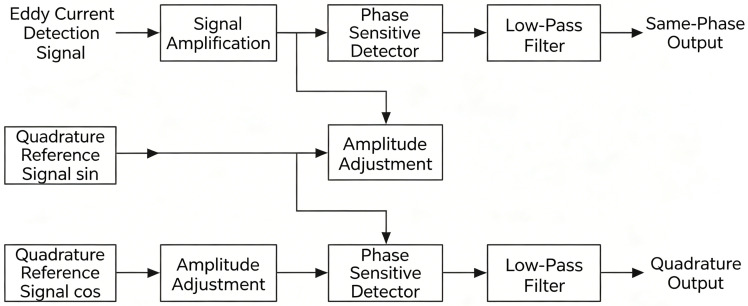
Signal processing procedure of the lock-in amplifier module.

The signal conditioning module includes an operational amplifier circuit and a lock-in amplifier circuit. Since the induced voltage in the receiving coil of the ACSM probe is a millivolt-level differential signal, the operational amplifier requires a high-precision differential instrumentation amplifier. This design employs the high-precision instrumentation amplifier AD620 as the post-stage amplifier. AD620 features proven performance, high accuracy, low power consumption, and compact size, meeting the detection system’s requirements for weak signal amplification and rapid processing of multi-channel data acquisition. The lock-in amplifier circuit extracts effective stress signals from noise-containing signals. This design uses the AD630 chip as the lock-in amplifier module, which contains a multiplier and a low-pass filter module. The signal processing procedure is shown in Figure 4. The DC signal output from the lock-in amplifier module can be mathematically processed to obtain the amplitude and phase information of the stress signal.

The A/D conversion module encodes the detected stress signal voltage values and converts them into digital quantities for convenient data processing by the main control chip. The main control chip employs the STM32F405RGT6 as the core embedded processor, with a main frequency of up to 168 MHz, exhibiting outstanding digital signal processing capability suitable for high-speed computation of stress signals. It integrates a hardware floating-point operation unit, enabling efficient floating-point calculations for faster and more accurate data processing.

**Figure 5:**
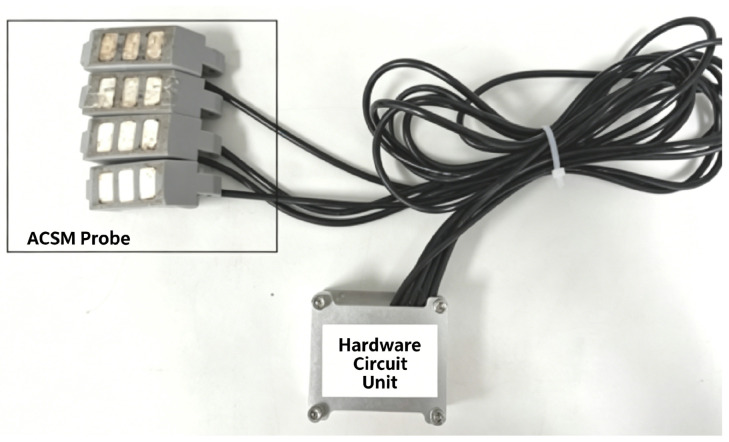
The ACSM detection system.

The probe module adopts an orthogonal differential coil group design, with manganese–zinc ferrite I-shaped magnetic cores inside the inductive coils to further concentrate and enhance the magnetic field. The integrated ACSM stress detection system is shown in Figure 5.

### 2.2. DIC Strain Detection Technology

Digital Image Correlation (DIC) technology reconstructs three-dimensional displacement and strain fields with hundreds of thousands of measurement points by comparing surface speckle images before and after object deformation, using computer algorithms to calculate full-field displacement and strain distributions with spatial resolution reaching the micrometer level. Since its inception in the 1980s, this technology has been widely applied in frontier fields such as aerospace composite material strength verification, biological soft tissue mechanical response analysis, and microelectronic packaging thermal deformation monitoring.

The DIC testing system achieves stress–strain detection through Hooke’s law and digital image correlation methods. It utilizes a high-resolution binocular vision system to capture the deformation of random speckle patterns on the surface of the measured material after loading, thereby deducing the strain state of the material.

Hooke’s law describes the relationship between the magnitude of deformation and the magnitude of external force when a material is in the elastic stage, as expressed in Equation (1). Within the elastic range, stress and strain exhibit a linear relationship, with the proportional coefficient determined by the material’s Young’s modulus:

*σ* = *E* × *ε* (1)

where *σ* is the material stress, *E* is the Young’s modulus of the material, and *ε* is the material strain.

The core of the digital image correlation method lies in the image matching algorithm, whose purpose is to find the region most similar to the sub-region in the reference image within the target image, thereby determining the surface displacement of the object. Commonly used core algorithms include cross-correlation algorithm, least-squares algorithm, and sub-pixel interpolation algorithm. The cross-correlation algorithm calculates the cross-correlation coefficient between the reference image subset and the target image subset to measure their similarity; a higher coefficient indicates greater similarity. The advantage of this algorithm is its computational simplicity and ease of implementation, though it is relatively sensitive to image noise and illumination variations. The least-squares algorithm minimizes the grayscale difference between reference and target image subsets to find the optimal matching position, offering better robustness against noise and illumination changes but requiring greater computational effort. The sub-pixel interpolation algorithm performs interpolation between pixel points to obtain more precise displacement values, with common techniques including bilinear and bicubic interpolation.

## 3. Finite Element Simulation of Axial Tensile Stress

### 3.1. Model Parameter Design

**Figure 6:**
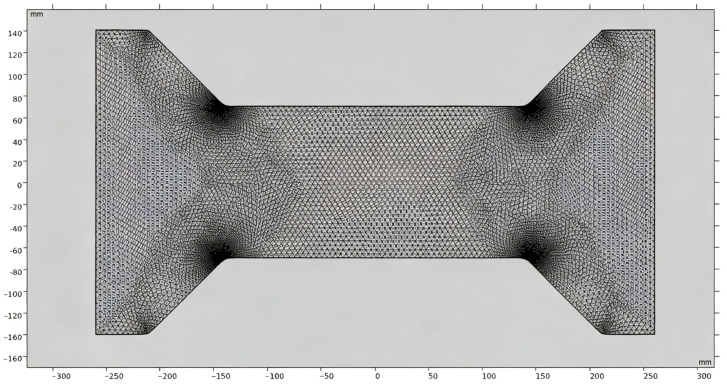
Two-dimensional model of the iron specimen.

Finite element simulation was conducted on the specimen to be used in the experiment. The specimen dimensions are shown in Figure 6, and the material performance parameters of the specimen are listed in Table 1.

COMSOL V6.1 finite element simulation software was employed to simulate the stress state of the specimen during the tensile process. Since the tensile loading of the iron specimen remains within the elastic range, the specimen body was set as a linear elastic material. A two-dimensional model of the same dimensions was established, as shown in Figure 6. The left side of the geometry was set as a fixed constraint end, and a uniform load was applied to the right side of the specimen in the positive *x*-axis direction.

### 3.2. FEM Simulation Results

Parametric scanning was performed on the load magnitude, with the load variation range set from 0 to 100 MPa and a step size of 10 MPa.

#### 3.2.1. Axial Tensile Stress Simulation Results

**Figure 7:**
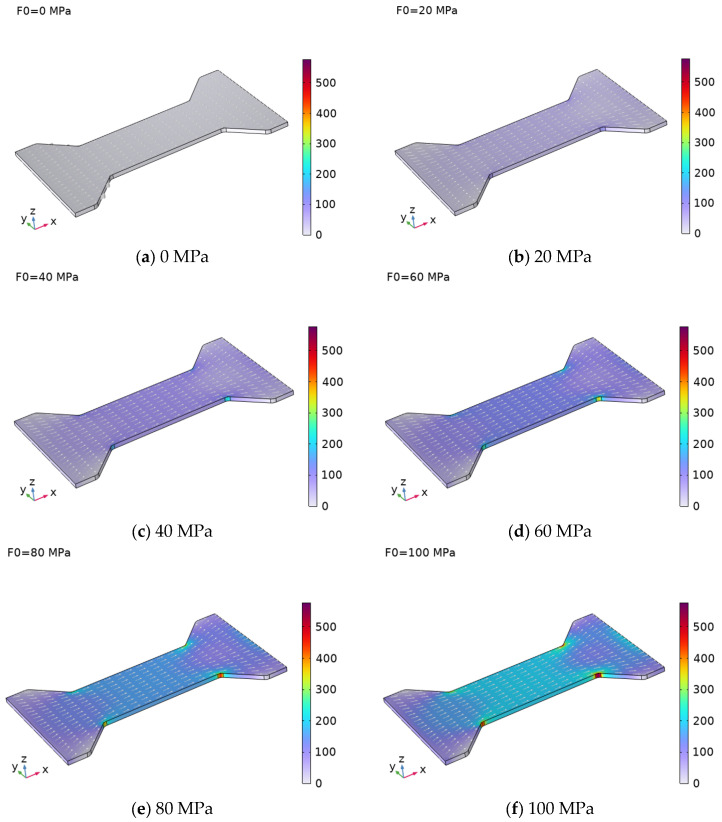
Von Mises stress contour map of the specimen surface.

The von Mises stress contour map extracted from the flat plate surface is shown in Figure 7. From the stress contour map, stress concentration occurs at the arc corners where the specimen transitions from the clamping section to the gauge section. Within the gauge section, the stress gradually increases from a smaller value to a fixed value and then stabilizes, with a uniform stress gauge section approximately 140 mm × 140 mm.

**Figure 8:**
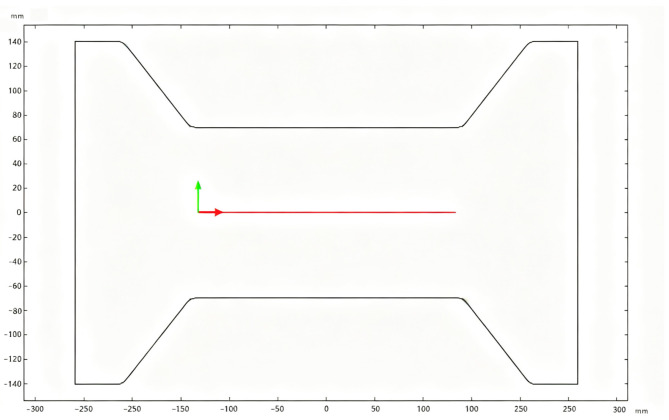
Line dataset extraction location on the specimen.

**Figure 9:**
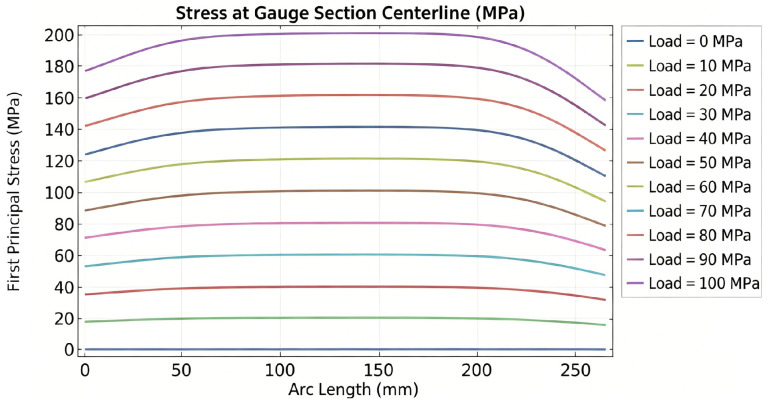
Stress variation along the arc length of the gauge section center line.

Along the length direction of the specimen, the center line of the equal cross-section rectangular region of the specimen was extracted as a line dataset, as shown in Figure 8, and the variation relationship of stress along the arc length was plotted, as shown in Figure 9. The axial stress generated in the gauge section is greater than the applied axial tensile load, with the ratio between the two approximating the ratio of the clamping section and gauge section cross-sectional areas. The axial stress in the gauge section is approximately twice the applied axial tensile load. The gauge section stress rises from both sides toward the middle and gradually stabilizes.

#### 3.2.2. Strain Simulation Results

**Figure 10:**
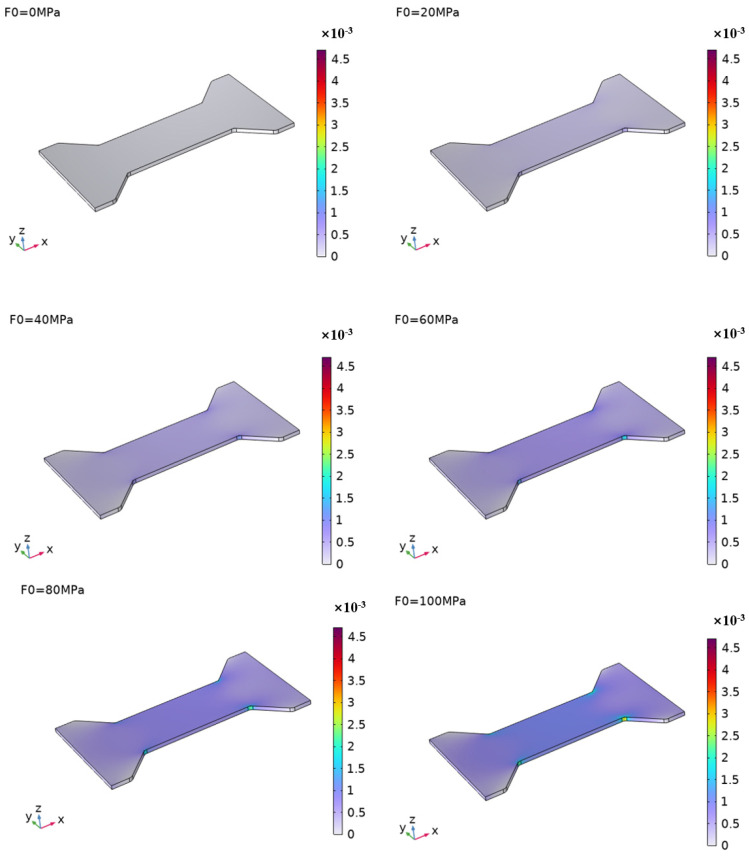
Full-field axial strain map.

**Figure 11:**
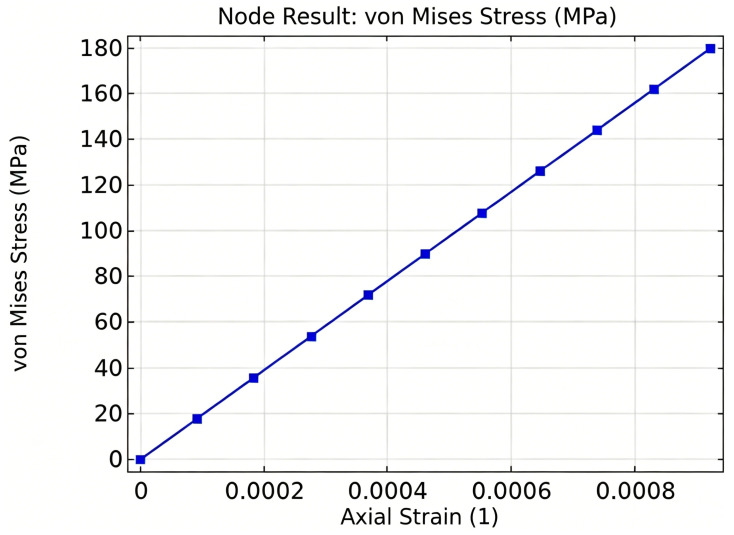
Stress–strain curve of the gauge section during the tensile process.

The full-field axial strain map was extracted, as shown in Figure 10. The axial strain maintains high uniformity within the gauge section; when the load is 100 MPa, the gauge section axial strain is approximately 9.24 × 10^−4^. In the clamping section, the axial strain distribution is similar to that of axial stress, with symmetric minimal value regions on both sides. The stress–strain curve of the gauge section throughout the entire tensile process is shown in Figure 11, with a curve slope of 200.01 GPa, approximating the simulation setting for Young’s modulus. Throughout the tensile process, the specimen remains in the elastic stage, satisfying Hooke’s law.

## 4. Experimental Research

### 4.1. Experimental System Setup

**Figure 12:**
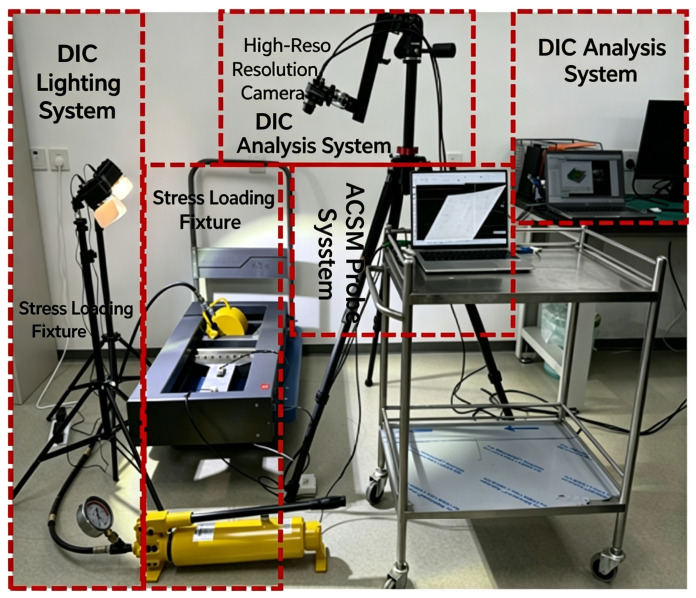
Photograph of the experimental system.

The experimental system comprises an iron specimen, a stress loading fixture, the ACSM stress detection system, and the DIC full-field strain detection system, as shown in Figure 12. The specimen dimensions are shown in Figure 12.

The ACSM stress detection system includes a self-developed ACSM stress testing probe, hardware circuit, and host computer. The DIC full-field strain detection system includes two high-resolution cameras, a camera support tripod, an illumination system, and a host computer. Since different materials have different Young’s moduli, the ACSM sensor that directly measures stress needs to be calibrated on the iron specimen first, obtaining the relationship curve between the corresponding voltage change and stress. Axial tensile loads from 0 to 100 MPa were applied to the specimen with a step size of 5 MPa, holding for 3 s at each step, while recording the corresponding loading force and ACSM sensor voltage output.

**Figure 13:**
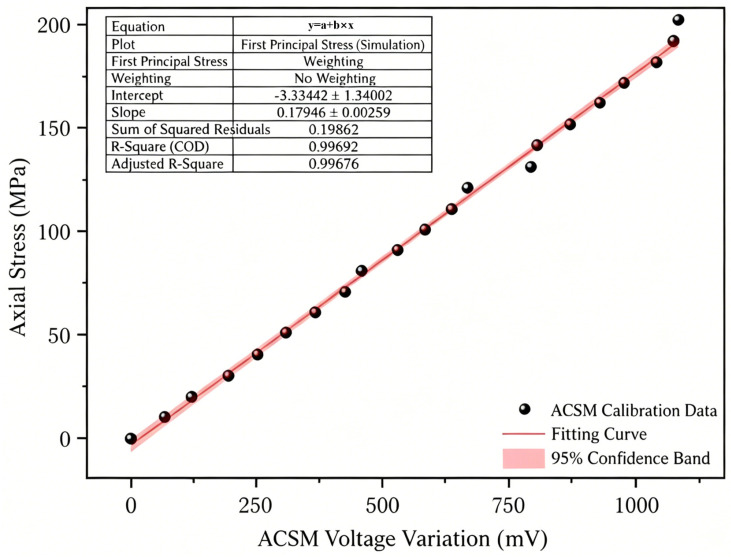
ACSM sensor voltage–axial stress fitting curve.

Linear fitting was used to calibrate the ACSM sensor, yielding the calibration equation:

(2) σ=0.17946×U−3.33442

where sigma represents the axial stress of the specimen to be tested (MPa), and *U* represents the voltage change in the ACSM sensor (mV). The fitted curve is shown in Figure 13, with residuals within +/−10 MPa at a confidence level of 95%.The data are shown in Table 2.

### 4.2. Experimental Process

The iron specimen was pre-treated with paint: first, an even layer of white matte paint was sprayed on the specimen surface; after the white paint solidified, a black random speckle pattern was printed onto it using a speckle roller. The iron specimen was fixed to the axial stress loading fixture test bench using bolts. The ACSM stress probe was attached to the left and right positions within the gauge section of the iron specimen using magnetically conductive adhesive, while the middle position of the specimen gauge section served as the DIC strain detection computation domain. Finally, the positions of the cameras and illumination system, along with camera parameters such as focal length and aperture, were adjusted to ensure that the painted area on the specimen surface could be clearly captured.

Before the experiment began, the DIC system was first calibrated using a calibration board. After calibration was completed, the calibration board was removed and the experiment commenced. Axial tensile loads from 0 to 100 MPa were applied to the specimen with a step size of 10 MPa, while simultaneously recording the axial loading force, ACSM system data, and DIC system data. The DIC system shooting frequency was set to 5 Hz.

### 4.3. Results and Analysis

**Figure 14:**
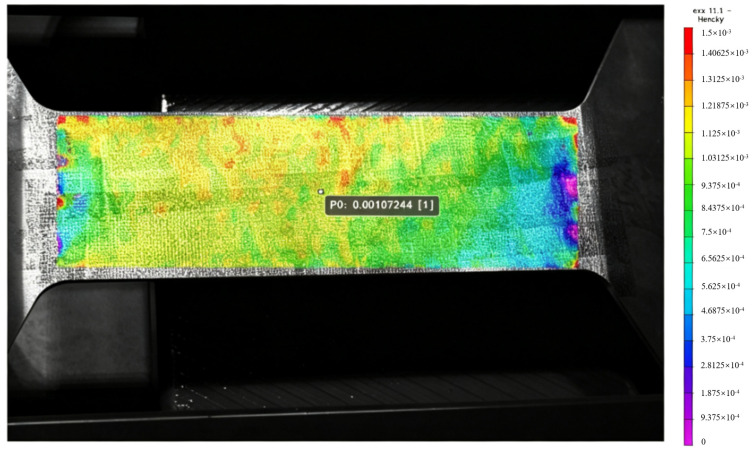
DIC full-field strain contour map.

The DIC data analysis system compares the specimen images obtained during the tensile process with the images captured during pre-tensile calibration, selecting feature points to calculate displacement and strain magnitudes. After filtering, the DIC full-field strain contour map is obtained, as shown in Figure 14. The strain distribution during specimen stretching is similar to the simulation results, with uniform strain distribution in the gauge section.

**Figure 15:**
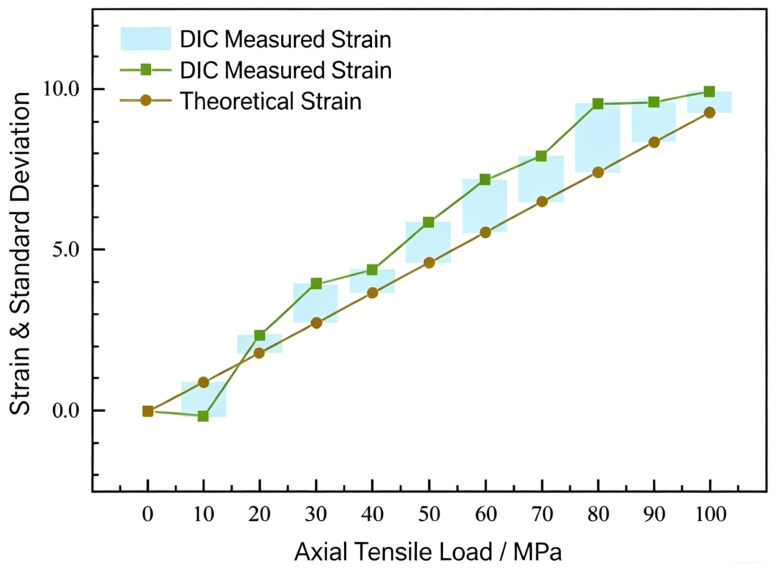
Error diagram of DIC full-field strain versus theoretical strain.

The DIC measured strain values, theoretical strain values, and their standard deviation during the tensile process are shown in Table 3. The error diagram of DIC strain versus theoretical strain is shown in Figure 15. At an axial tensile load of 80 MPa, the absolute error between the DIC measured strain value and the theoretical strain value is maximum, at 0.000214722. The relative error is maximum at an axial tensile load of 10 MPa, but as the tensile load increases, the relative error shows a decreasing trend. This indicates that the DIC strain testing system has a certain systematic error.

**Figure 16:**
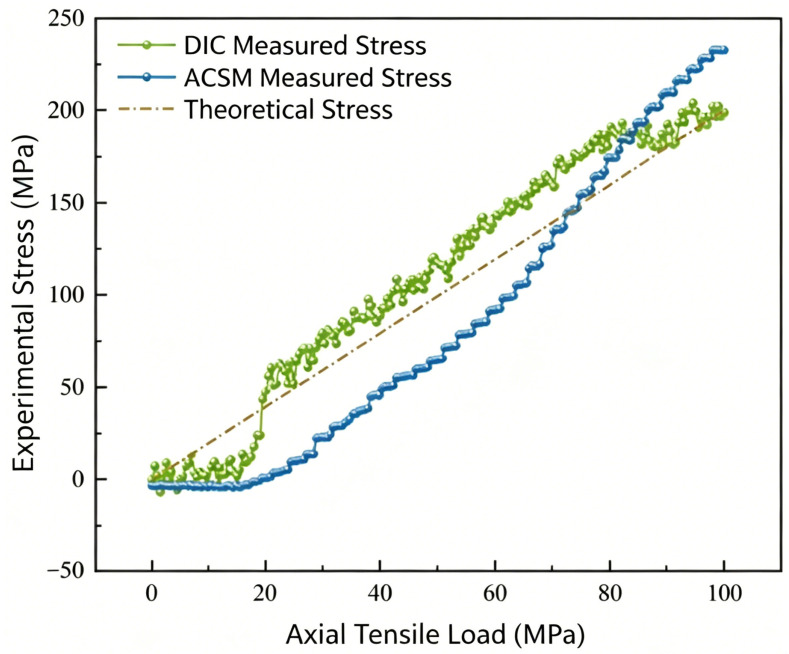
Comparison diagram of stress data during the tensile process.

Using the DIC measured strain data during the tensile process, the corresponding stress data was calculated and compared with the theoretical stress and ACSM measured stress data, as shown in Figure 16. Before 25 MPa, the strain values measured by both DIC and ACSM showed minimal changes, indicating insufficient sensitivity for detecting small stress variations. After 25 MPa, the stress values measured by DIC approach the theoretical stress values, with a maximum error of 31 MPa at an axial tensile load of 70 MPa.

**Figure 17:**
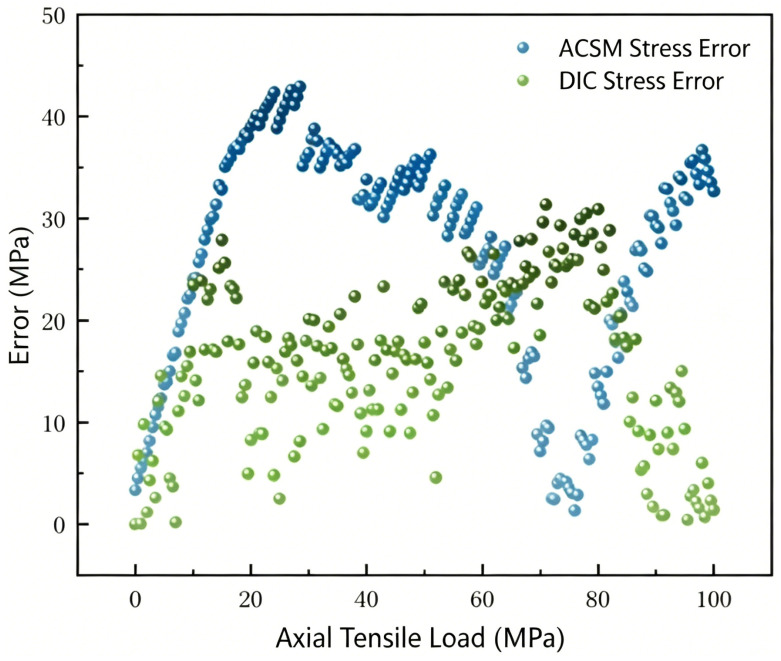
Stress error comparison diagram.

Compared with DIC, the ACSM stress data shows larger errors relative to the theoretical stress data, but the errors are primarily concentrated in the small stress range below an axial tensile load of 50 MPa. At approximately 28 MPa, the ACSM measured stress value exhibits the maximum absolute error of 42.93 MPa relative to the theoretical value, as shown in Figure 17.

## 5. Conclusions

This study, addressing the demand for non-destructive stress–strain testing of ferromagnetic components, systematically conducted a performance comparison and experimental verification of the ACSM method and the DIC technique. Combined with finite element numerical simulation and axial tensile tests, the detection accuracy and error characteristics of the two techniques were quantified. The following core conclusions are drawn:

(1) During the axial tensile process of the flat plate specimen, stress concentration predominantly occurs in the arc transition regions between the clamping section and the gauge section. A region of approximately 140 mm × 140 mm with uniformly distributed stress and strain exists in the central part of the gauge section. The simulation results provide a reliable numerical basis for the arrangement of measurement points and data calibration in the experiments.
(2) The self-developed ACSM stress detection system, after linear calibration, exhibits stable detection performance. At a 95% confidence level, the residuals of the calibration fit are controlled within ±10 MPa. Over the full load range, the maximum absolute detection error is 42.93 MPa, with the errors concentrated in the low-stress range below 50 MPa. In the medium-to-high stress range, the linearity and stability of the measurements are significantly improved, demonstrating that the system meets the accuracy requirements for rapid stress screening in industrial field applications.
(3) With reference to the theoretical solutions, the maximum absolute error of strain measurement by the DIC system is 2.14722 × 10^−4^, and the maximum stress error derived via Hooke’s law inversion is 31 MPa, showing good agreement with the theoretical true values. DIC enables simultaneous acquisition of full-field strain distributions and can intuitively present the spatial distribution characteristics of stress and strain within the gauge section. This provides a reliable full-field reference benchmark for the selection of measurement points, system calibration, and error correction in the single-point ACSM detection.

## Figures and Tables

**Table 1:** Material parameters of the simulation model.

Parameter	Value	Unit
Young’s Modulus (E)	200	GPa
Density	7850	kg/m^3^
Poisson’s Ratio	0.3	-
Yield Strength	250	MPa

**Table 2:** ACSM calibration data.

Axial Tensile Load (MPa)	Theoretical Axial Stress (MPa)	ACSM Voltage Variation (mV)
0	0	0
5	10.08831	66
10	20.17661	120
15	30.26492	195
20	40.35322	250
25	50.44153	306
30	60.52983	365
35	70.61814	424
40	80.70644	457
45	90.79475	530
50	100.8831	585
55	110.9714	639
60	121.0597	670
65	131.148	795
70	141.2363	807
75	151.3246	871
80	161.4129	930
85	171.5012	979
90	181.5895	1044
95	191.6778	1076
100	201.7661	1086

**Table 3:** Strain data during the axial tensile process.

Load (MPa)	DIC Strain	Theoretical Strain	Relative Error (%)
10	0	0	0
20	−1.71027 × 10^−5^	9.24482 × 10^−5^	1.09551× 10^−4^
30	2.41231× 10^−4^	1.84896× 10^−4^	5.6335× 10^−5^
40	4.00497× 10^−4^	2.77345× 10^−4^	1.23152× 10^−4^
50	4.45427× 10^−4^	3.69793× 10^−4^	7.5634× 10^−5^
60	5.89029× 10^−4^	4.62241× 10^−4^	1.26788× 10^−4^
70	7.18774× 10^−4^	5.54689× 10^−4^	1.64085× 10^−4^
80	7.92628× 10^−4^	6.47137× 10^−4^	1.45491× 10^−4^
90	9.54307× 10^−4^	7.39585× 10^−4^	2.14722× 10^−4^
100	9.60529× 10^−4^	8.32034× 10^−4^	1.28495× 10^−4^

## Data Availability

The data presented in this study are available on request from the corresponding author.
